# Case Report: A case of rapamycin-eluting stent for the treatment of refractory stenosis of arteriovenous fistula stenosis

**DOI:** 10.3389/fcvm.2024.1449989

**Published:** 2024-08-27

**Authors:** Yu Xiong, Bo Tu, Minglu Zhang, Bo Chen, Qiquan Lai, Jing Chen, Ling Chen, Ziming Wan

**Affiliations:** ^1^ Department of Nephrology, The First Affiliated Hospital of Chongqing Medical University, Chongqing, China; ^2^ Department of Ultrasonography, The First Affiliated Hospital of Chongqing Medical University, Chongqing, China; ^3^ Department of Ultrasonography, The 941st Hospital of the PLA Joint Logistic Support Force, Xining, China; ^4^ Department of Nephrology, Southwest Hospital Jiangbei Area (The 958th Hospital of Chinese People’s Liberation Army), Chongqing, China

**Keywords:** arteriovenous fistula, refractory stenosis, rapamycin-eluting stent, percutaneous transluminal angioplasty, end-stage renal disease

## Abstract

For patients with repeated stenosis of autologous arteriovenous fistula, percutaneous transluminal angioplasty (PTA) or bare metal stent placement had limited efficacy. Rapamycin was reported to inhibit neointimal hyperplasia and keep blood vessels patent. In this study, we reported a case with refractory stenosis, i.e., a short duration of patency maintenance after each repeated PTA, which was treated with a rapamycin-eluting stent (RES). The RES extended the patency duration from 4 to 5 months on average to 14 months. The stent was used to maintain dialysis for over 30 months. RES may be an effective way to treat refractory stenosis and salvage limited vascular resources.

## Introduction

Autologous arteriovenous fistula (AVF) is the preferred vascular access for patients with end-stage renal disease (ESRD), accounting for 80.5% of all vascular access (1), and is also known as the “lifeline” of dialysis patients. However, AVF is prone to restenosis due to various reasons, and neointimal hyperplasia is the most common one (2). It was reported that the 2-year patency rate of AVF is only 38%–56% (3). Percutaneous transluminal angioplasty (PTA) is an effective way to deal with AVF restenosis (4). However, a few patients may experience repeated stenosis in the short term. For these patients, repeated PTA or bare metal stent placement had limited efficacy.

Drug-eluting stents (DESs) can effectively reduce the risk of vascular restenosis through coated antiproliferative drugs, such as rapamycin or paclitaxel, which can be slowly released into the surrounding tissues to inhibit neointimal hyperplasia and keep blood vessels patent. In addition, the stent itself can provide long-term vascular support for maintaining patency. DESs have been proven to be effective in the treatment of restenosis in patients with percutaneous coronary intervention (5). However, the literature on DESs for treating repeated stenosis of AVF in ESRD patients is limited, especially for rapamycin-eluting stents (RESs). Here, we reported a case of using an RES to treat refractory stenosis of AVF, which resulted in a long-term patency duration.

## Case presentation

A 51-year-old female patient was diagnosed with chronic kidney disease (stage 5) which was caused by polycystic kidney disease. This patient had a history of hypertension and no other complications, such as diabetes or coronary heart disease. Furthermore, she had no history of smoking or drinking wine. Her body mass index was 18.47. This patient had received hemodialysis since 2014. On 10 November 2015, an autologous AVF in the right wrist was created in our hospital which was occluded 1 month later. Then, we reconstructed an AVF in the right forearm during open surgery on 30 December 2015. This AVF was used for hemodialysis for approximately half a year and was occluded again. An ultrasound-guided PTA was performed to clear the occlusion on 29 June 2016, with a balloon of 5 mm × 40 mm. Shortly thereafter, this patient underwent replete angioplasty because of repleted stenosis in right forearm AVF on 11 October 2016, and 7 February 2017, with a balloon of 6 mm × 40 mm.

**Figure 1:**
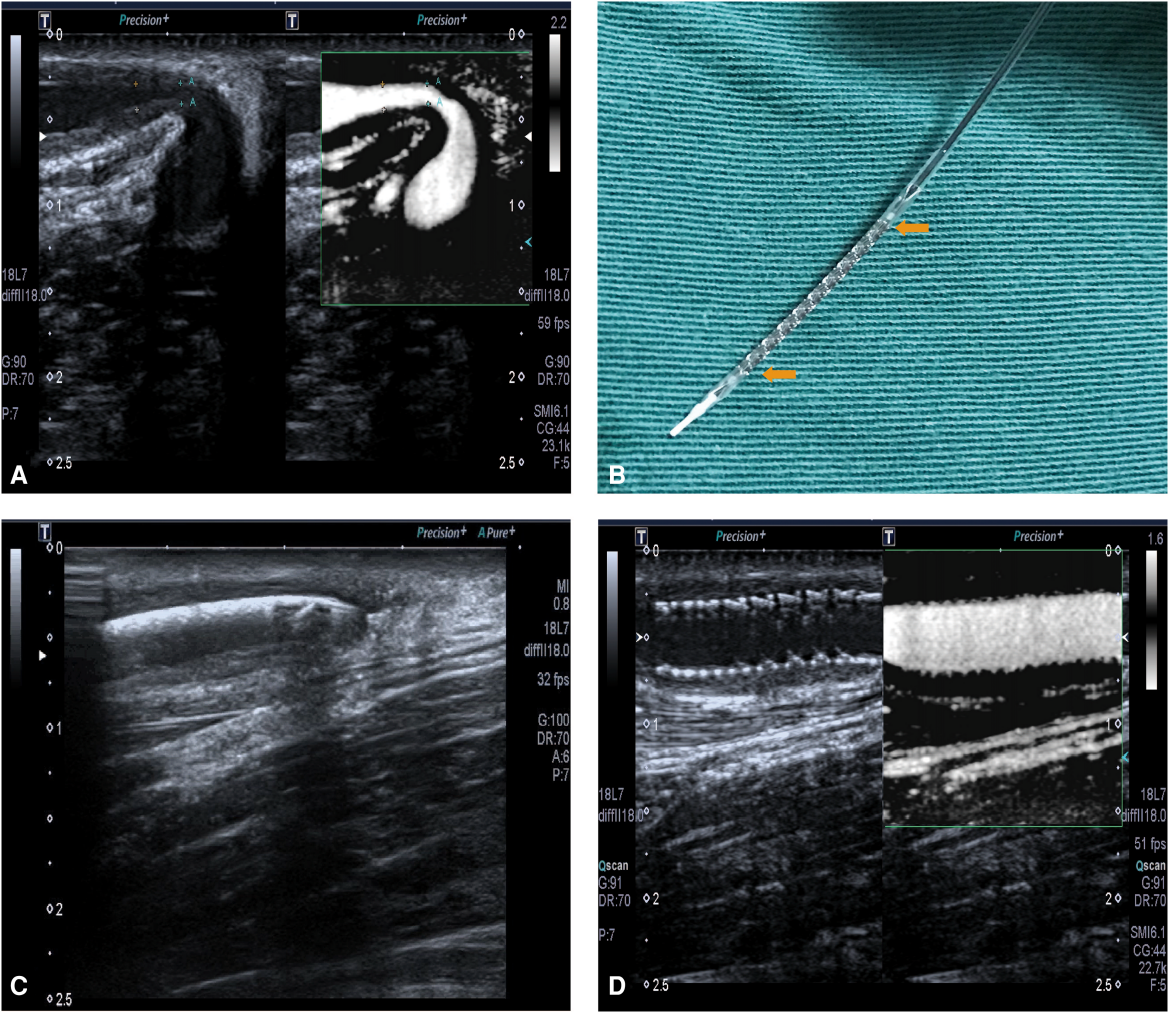
**(A)** The restenosis of right forearm autologous AVF on 23 May 2017. **(B)** RES. The yellow arrows show the main body of the stent, with a balloon inside it. **(C)** The ultrasound localization of RES which shows the RES was placed at the site of stenotic lesion. **(D)** The fully expanded RES, with a patent blood flow.

On 23 May 2017, this patient was hospitalized again because of decreased flow volume in the right forearm AVF. Preoperative ultrasonoscopy indicated a significant juxta anastomotic stenosis, with a diameter of 1.0 mm and a length of 1.5 cm (Figure 1A). No thrombosis or calcification was found. Because of the short duration of patency maintenance after each repeated PTA, after a discussion, the team decided to perform another PTA with RES placement in the right forearm AVF. Informed consent was obtained before the procedure.

### Procedure

After the brachial plexus block, a 5F-R vascular sheath (Terumo, Tokyo, Japan) was inserted into the cephalic vein. A guide wire (Abbott Vascular, Santa Clara, California, USA) of 0.014 in. was advanced along the vein slightly across the stenotic lesion. A balloon of 5 mm × 20 mm was advanced along the guide wire and across the stenotic lesion to the anastomotic site, followed by balloon angioplasty of the stenotic lesion, the outflow vein, and the anastomotic site using 20–24 atm, repeated three times. The balloon was subsequently removed. Then the RES (NOYA, Beijing, China), a balloon-expandable stent with a diameter of 4.5 mm and length of 22 mm, was placed in the stenotic lesion with the guidance of ultrasonography (Figures 1B,C). The RES was fully expanded by a balloon angioplasty using 16 atm.

After PTA and RES placement, the ultrasound images showed that the diameter of the brachial artery was 5.9 mm, with a flow volume of 521 ml/min. In the segment of the cephalic vein near an anastomosis, a 22 cm stent was observed. The stent was closely fitted to the vascular wall, and a color Doppler ultrasound showed filled blood flow through the stent, with a peak velocity of 210 cm/s (Figure 1D). The diameter of the middle segment of the forearm cephalic vein was 3.8 mm, with an intimal medial thickness of 1.4 mm and a peak velocity of 256 cm/s.

**Figure 2:**
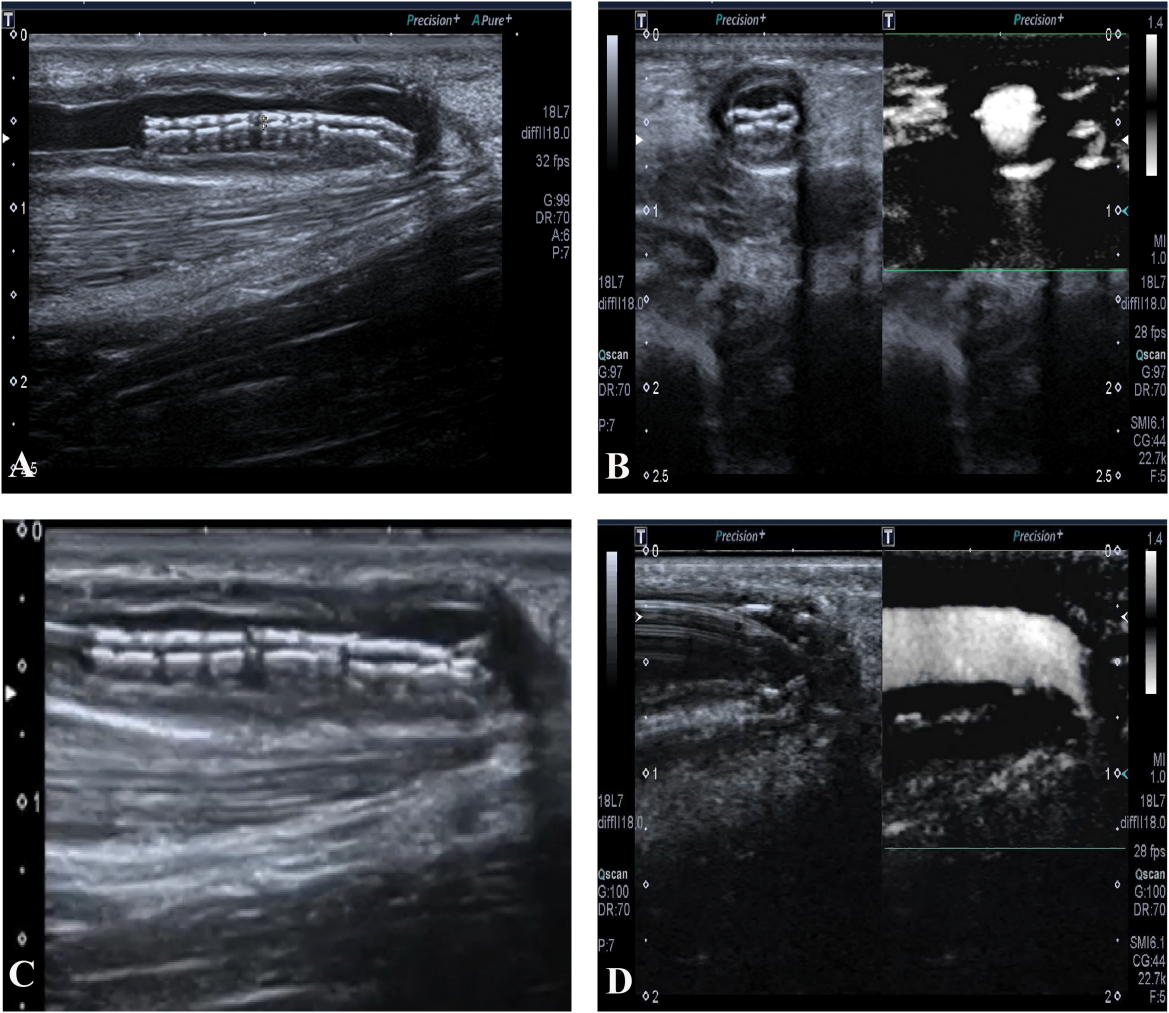
**(A)** The collapsed stent in long-axis view. **(B)** The collapsed stent in short-axis view, with acceptable blood flow. **(C)** The guide wire advances through the collapsed stent. **(D)** The expansion of the collapsed stent using percutaneous transluminal angioplasty.

The right forearm AVF was patent for hemodialysis until the stent collapsed on 27 July 2018, with a primary patent duration of 14 months. No other postoperative complications occurred before the stent collapsed, such as thrombosis, heart failure, pseudoaneurysm, infection, or hemodialysis access-induced distal ischemia syndrome. Though the flow volume was acceptable, we still decided to expand the stent using PTA (Figure 2). On 30 July 2019, another restenosis occurred in the proximal part of the outflow vein of the right forearm AVF and was treated using ultrasound-guided PTA. On 26 December 2019, a thrombosis related to the restenosis in the proximal part was found by ultrasonography. Hence, the right forearm AVF was discarded. Instead, an arteriovenous graft (AVG) in the left forearm was constructed for maintenance hemodialysis since then, with one PTA procedure annually on average up to now.

## Discussion

Though DESs and drug-eluting balloons have been proven to be effective in treating restenosis in patients with acute cardiovascular events (6), the evidence for using DESs to treat refractory stenosis in AVF patients is lacking. A pilot study reported the efficacy of a paclitaxel-eluting stent (PES) for treating AVF dysfunction. With a mean follow-up of 202 days, seven out of nine patients had patent AVFs (7). A case report reported that the PES significantly extended the interval time of repeated balloon angioplasty from 3.1 to 5 months (8). A study demonstrated that among 14 patients who received a PES, the primary patency rates at 6 and 12 months were 64% and 29%, respectively, which were more pronounced than that of their last conventional balloon angioplasty (29% and 7%, respectively) (9). All these studies demonstrated effective improvement when using a PES to treat frequent restenosis.

To the best of our knowledge, no study has reported the use of an RES for treating frequent restenosis in AVF patients. In this study, the RES extended the patency duration from 4 to 5 months on average to 14 months. The stent was used to maintain dialysis for over 30 months. Furthermore, the stent collapsed only once and another restenosis occurred in the meantime.

Rapamycin, also known as sirolimus, is a macrolide antibiotic class immunosuppressant. It inhibits the degradation of the cyclin-dependent kinase inhibitor (p27), thereby suppressing the G1 phase of the cell cycle and preventing cells from entering the next cycle (10). This mechanism explains its anti-smooth muscle cell proliferation properties and ability to inhibit thrombosis formation on stents. In addition, rapamycin can block the synthesis of proteins required for cell division, and its binding to FKBP12 weakens mTOR activity, further suppressing the cell cycle (11).

Another strength of this case is the guidance by ultrasonography, which helped monitor the position of the stent in real-time, and clearly showed the shape of the stent and its adhesion to the blood vessel wall. In addition, ultrasonography had the advantage of allowing us to evaluate the hemodynamics after stent placement and subsequently monitor the long-term patency.

A study reported that a balloon-expandable stent is vulnerable to external compression and this can result in collapse. This may be the main reason for the collapse of our case as the RES was placed in a cephalic vein, a superficial anatomy (12). In addition, rough operation may also lead to a collapse, which suggests experience and gentle handling are needed (13).

In conclusion, ultrasound-guided rapamycin-eluting stent placement is effective for treating refractory stenosis of AVF, with acceptable adverse events and long-term patency. This may be an effective way to salvage limited vascular resources.

## Data Availability

The raw data supporting the conclusions of this article will be made available by the authors, without undue reservation.
